# TRPV4 activation prevents lipopolysaccharide-induced painful bladder hypersensitivity in rats by regulating immune pathways

**DOI:** 10.3389/fimmu.2022.1080302

**Published:** 2022-12-22

**Authors:** Masaru Yoshizumi, Naoya Tazawa, Chizuko Watanabe, Hirokazu Mizoguchi

**Affiliations:** Division of Physiology and Anatomy, Faculty of Pharmaceutical Sciences, Tohoku Medical and Pharmaceutical University, Sendai, Japan

**Keywords:** TRPV4, LPS, interstitial cystitis, bladder pain syndrome, anti-inflammation

## Abstract

Chronic inflammation in the urinary bladder is a potential risk factor for bladder dysfunction, including interstitial cystitis/bladder pain syndrome (IC/BPS). Although several studies have reported that activation of transient receptor potential vanilloid 4 (TRPV4) contributes to bladder pain and overactive bladder with a cardinal symptom of acute or chronic cystitis, others have reported its involvement in the protective response mediated by lipopolysaccharides (LPS) to secrete anti-inflammatory/pro-resolution cytokines. Therefore, we investigated the potential benefit of an intravesical TRPV4 agonist for painful bladder hypersensitivity in a rat model of LPS-induced cystitis and determined whether its effects modulate the LPS signal for inflammatory reaction, cytokine release, and macrophage phenotype change. Previously, we showed that repeated intravesical instillations of LPS induce long-lasting bladder inflammation, pain, and overactivity in rats. In the present study, concurrent instillation of the selective TRPV4 agonist GSK1016790A (GSK) with LPS into the rat bladder improved LPS-induced bladder inflammation and reduced the number of mast cells. Furthermore, co-instillation of GSK prevented an increase in bladder pain-related behavior and voiding frequency caused by LPS. Cytokine profiling showed that LPS-stimulated inflammatory events, such as the production and secretion of pro-inflammatory cytokines (CXCL1, CXCL5, CXCL9, CXCL10, CCL3, CCL5, CCL20, and CX3CL1), are suppressed by GSK. Furthermore, TRPV4 activation switched LPS-stimulated pro-inflammatory M1-type macrophages to anti-inflammatory M2-type macrophages. These results suggest that TRPV4 activation in the bladder negatively regulates the pro-inflammatory response induced by LPS and prevents bladder hypersensitivity. These TRPV4 functions may be promising therapeutic targets for refractory IC/BPS.

## Introduction

Interstitial cystitis/bladder pain syndrome (IC/BPS) is a multifactorial, chronic bladder disorder with cardinal symptoms of pain in the bladder and pelvis and lower urinary tract symptoms, such as urinary frequency and urgency (1, 2), often associated with other painful diseases that profoundly affect patients’ quality of life (3). Although IC/BPS typically has no bacteriuria, epidemiological evidence of the disease supports past tract infections as a risk factor (4). Our recent study in rats showed that repeated intravesical instillation of *Escherichia coli*-derived lipopolysaccharides (LPS) results in a model of chronic cystitis with persistent bladder pain and urinary frequency (5). Therefore, IC/BPS could still be a sterile/persistent inflammation state initiated by initial microbial cystitis.

Transient receptor potential vanilloid 4 (TRPV4), a non-selective cation channel of the TRP superfamily (6), has been implicated in several physiological processes. TRPV4 is commonly considered a mechano- or osmo-sensor (7–10) and can also be activated by moderate heat (11, 12). Therefore, TRPV4 indicates constitutive activity at physiological temperatures and an up-regulation in fever and local temperature rise; thus, it responds to inflammation by various chemical stimuli (6, 13, 14) and acts as a pathological sensor.

TRPV4 expression has been shown in the bladder epithelium and is implicated in the regulation of urothelial ATP release, which modulates the sensitivity of bladder afferent nerves (15–17). Intravesical instillation of GSK1016790A (GSK), a TRPV4 potent agonist, induces bladder hyperactivity in rodents and increases the firing of afferent bladder nerves (18, 19). Conversely, TRPV4 knockout (KO) mice or pharmacological blockade of TRPV4 improved voiding frequency and decreased void volume after LPS- or cyclophosphamide (CYP)-induced bladder inflammation (20, 21). Recent work has reported that TRPV4 is directly involved in bladder pain induced in CYP cystitis model and provides an analgesic effect by blocking TRPV4 (22).

Interestingly, some *in vitro* studies have demonstrated that TRPV4 mediates LPS signaling and alters the cytokine response to anti-inflammation/pro-resolution (21, 23–25). Furthermore, in airway or bladder epithelial cells, LPS triggers a potent protective response *via* TRPV4 activation in a canonical Toll-like receptor 4 (TLR4) immune pathway-independent manner (21, 24). However, the resolution of inflammatory pain owing to the protective role of TRPV4 in cystitis-associated painful bladder hypersensitivity *in vivo* remains to be elucidated. In this study, we sought to determine whether TRPV4 activation alleviates the LPS signaling-mediated inflammatory reaction and modulates cytokine release and macrophage phenotypic changes, thereby improving painful bladder hypersensitivity. This study is potentially applicable to bladder defense against infection and the resolution of chronic inflammation, such as IC/BPS.

## Materials and methods

### Animals

Adult female 8–9-week-old Sprague-Dawley rats (Japan SLC, Hamamatsu, Japan) weighing 200–250 g were used in this study. The animals were housed in a room maintained at 22–24°C and 50–60% relative humidity with an alternating 12-h light–dark cycle. Food and water were provided *ad libitum*. All animal procedures were approved by the Committee of Animal Experiments, Tohoku Medical and Pharmaceutical University, and performed in accordance with the NIH Guide for the Care and Use of Laboratory Animals.

### Induction of LPS-induced cystitis

**Figure 1:**
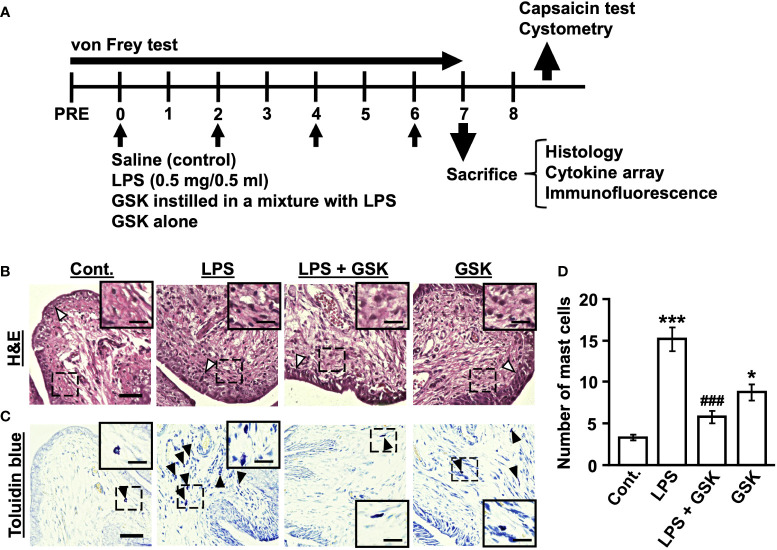
GSK reduced LPS-induced inflammatory reaction and the number of mast cells in rat bladder. **(A)** Schematic diagram of the experiments in chronic cystitis rats. The bladders of rats were instilled with LPS (0.5 mg/0.5 ml in vehicle) or the selective TRPV4 agonist GSK1016790A (GSK) in a mixture with LPS once every 48 h for 4 days. **(B)** Photomicrographs of hematoxylin and eosin (H&E) staining in rat bladder samples. The thickness of bladder epithelial cell indicated by white arrows. **(C)** Representative photomicrographs of rat bladder samples stained with toluidine blue (black arrows) showing mast cells. Magnification 40× (object lens), scale bars, 50 μm. The boxed region from the merge is enlarged in the inset (scale bars, 20 μm). **(D)** Plot showing the number of mast cells in the bladder of rats. Data represent the mean ± SEM; *n* = 4 for each group. *P < 0.05, ***P < 0.001 vs control. ^###^ *P* < 0.001 vs LPS.

The rats were anesthetized with 2% isoflurane (Pfizer Inc., New York, NY, USA). A PE-50 polyethylene tube (Becton Dickinson, Sparks, MD, USA) was inserted into the bladder *via* the urethra to empty the bladder, and 0.5 ml of LPS (*E. coli* O55:B5, Sigma-Aldrich, St. Louis, MO, USA) at 1 mg/ml in sterilized saline was infused intravesically and remained in the bladder for 30 min. After LPS exposure, the bladder was rinsed once with saline and allowed to drain freely from the open catheter end. Chronic cystitis was induced by intravesical LPS administration once every 48 h for 4 times (**Figure 1A**). The control group rats were received administered 0.5 ml of saline into the bladder.

### Drugs and administration

GSK1016790A (GSK) was purchased from FUJIFILM Wako Pure Chemical Corporation (Osaka, JAPAN). Stock solutions of GSK were dissolved in 100% DMSO and adjusted with saline to a final concentration of 0.1%. Intravesical GSK was instilled in a mixture with LPS using the same protocol, as shown in **Figure 1A**.

### Histology

Bladders were isolated from the rats in all groups and fixed in 4% paraformaldehyde. Tissues were frozen and cut on a cryostat at a thickness of 5-μm parallel to the equatorial plane, and then stained with hematoxylin and eosin (H&E) and toluidine blue for visualizing inflammatory cell infiltration and mast cells, respectively. Three random areas per bladder section from each group were observed and the number of mast cells in the lamina propria and muscle layer was estimated within the visual fields observed at object lens 20× magnification under an all-in-one fluorescence microscope (BZ-X800; KEYENCE, Osaka, Japan).

### Behavioral studies

#### von Frey test

Hypersensitivity responses to the lower abdomen and hind paw were assessed using calibrated von Frey filaments (Danmic Global LLC, San Jose, CA, USA). The rats were placed individually in a small acrylic cage with a wire mesh floor and acclimated to the experimental environment for 1 h. Eight von Frey filaments exerting 2-60 g were used for the abdominal stimulation to assess the pain threshold. The tactile sensitivity of the region between the anus and urethral opening was assessed by applying filaments perpendicular to the surface of the skin. Repeated stimulation of the same location was avoided to prevent the wind-up effects of desensitization. Behaviors considered positive responses to filament stimulation were sharp retraction of the abdomen, immediate licking or scratching of the area of filament stimulation, and jumping. For hind paw stimulation, eight von Frey filaments exerting 0.6-26 g were used to assess the pain threshold. Filaments were applied to the plantar surface of the hind paw, and brisk paw withdrawal was considered as positive response. The withdrawal threshold was determined using the up-down statistical method (26).

#### Capsaicin test

Before testing, the rats were placed in individual transparent plastic cages (35 cm × 40 cm × 18 cm) for at least 30 min to adapt to the novel environment. After anesthesia with isoflurane, a PE-50 polyethylene tube (Becton Dickinson) was inserted into the bladder through the urethra, and residual urine was withdrawn. Rats were placed in a Ballman restraining cage (Natsume Seisakusho, Tokyo, Japan) and allowed to recover from anesthesia. Capsaicin solution or vehicle was then instilled into the bladder *via* the catheter (0.5 ml and kept for 30 s). Subsequently, the transurethral catheter was removed, and the rats were placed in a transparent plastic cage. Bladder pain-related lower abdominal licking behavior was measured for each rat over 20 min.

### Cystometry

Cystometry was performed as we had reported previously (5). The rats were anesthetized using 2% isoflurane, and a midline abdominal incision was made to expose the bladder. A PE-50 polyethylene tube with a fire-flared tip was implanted into the bladder dome for bladder filling and pressure recording two days before the experiments. The catheter was then implanted in the subcutaneous space. In cystometry, rats were placed in a Ballman restraining cage (Natsume Seisakusho) and the indwelling catheter to the bladder was connected *via* a three-way stopcock to a pressure transducer and a syringe pump (KD Scientific Inc., Holliston, MA, USA). Physiological saline was infused at room temperature (22–24°C) into the bladder at a rate of 2.4 ml/h. The intravesical pressure was recorded using a force transducer, quad bridge amplifier FE224 (ADInstruments, Bella Vista, NSW, Australia), and PowerLab data-acquisition system with LabChart Pro (ADInstruments).

### Cytokine array

Bladder tissues were homogenized, lysed, and centrifuged for 10 min at 4°C at 2,000 × g. Protein content in each supernatant was measured using a standard Bradford method. The cytokine array in the harvested samples was analyzed using the Rat Cytokine Array Panel A kit from R&D Systems (Minneapolis, MN, USA). The samples were then diluted and mixed with a cocktail of biotinylated antibodies. The sample/antibody mixture was incubated overnight at 4°C with a rat cytokine array membrane. After several washes, the membranes were briefly incubated with streptavidin conjugated to horseradish‐peroxidase (HRP). The membranes were then washed several times, exposed to chemiluminescent detection reagents, and detected using the luminescent image analyzer ImageQuant LAS4000 (GE Healthcare, Chicago, IL, USA). The density of each spot was measured using a computer-assisted imaging analysis system (ImageQuant TL). The negative control spots on the array were selected as the background, and relative changes in cytokine expression levels were determined by normalizing the conditions to the control condition.

### Immunofluorescence analysis

Bladders were isolated from the rats in all groups and fixed in 4% paraformaldehyde. The tissues were frozen and cut on a cryostat at 5-μm thickness. Sections were blocked with 5% bovine serum albumin in Tris-buffered saline containing 0.1% Tween 20 for 1 h at room temperature. The slides were incubated with mouse anti-F4/80 (1:50; Santa Cruz Biotechnology Inc., Dallas, TX, USA), rabbit anti-iNOS (1:100; Abcam plc, Cambridge, UK), or rabbit anti-CD206 (1:500; Abcam plc) overnight at 4°C and then with anti-mouse Alexa Fluor Plus 594 (1:1000; Abcam plc), anti-rabbit Alexa Fluor Plus 488 (1:1000; Thermo Fisher Scientific, Waltham, MA, USA) secondary antibodies, and 4′,6-diamidino-2-phenylindole (DAPI; Thermo Fisher Scientific) for 1 h at room temperature. Immunofluorescence images were obtained using an all-in-one fluorescence microscope (BZ-X800; KEYENCE) and analyzed using a BZ-X800 analyzer. Two random areas per bladder selection from each group were observed and the macrophage in the lamina propria was estimated within the visual fields observed at object lens 10× magnification. The percentage of iNOS- or CD206-positively stained cells per F4/80-positively stained cells in the fields was determined.

### Statistical analysis

Data are presented as the means ± SEM. Differences in the withdrawal threshold between the groups were analyzed using one- or two-way analysis of variance (ANOVA) followed by either Dunnett’s test or Tukey’s test. GraphPad Prism version 7 (GraphPad Software, San Diego, CA, USA) was used for statistical analysis. Statistical significance was set at *P* < 0.05. A parametric test (unpaired t-test) was used to test for differences in the cystometric variables between the two groups.

## Results

### TRPV4 alleviates the LPS-induced bladder pathology

In the bladder tissue from the LPS-instilled group, thickening of the urothelium and inflammatory cell infiltration in the submucosal layers were observed compared with the control group (**Figure 1B**). This situation was improved by the co-instillation of GSK (10 μM) with LPS (**Figure 1B**). However, GSK alone was associated with thickening of the urothelium and inflammatory cell infiltration (**Figure 1B**). The mast cell count in the LPS + GSK group was significantly lower than that in the LPS group but was not significantly different from that in the control group (**Figures 1C, D**). In the GSK alone group, the number of mast cells was significantly higher than that in the control group and lower than that in the LPS group (**Figures 1C, D**).

### TRPV4 inhibits the development of LPS-induced bladder pain-related behavior

**Figure 2:**
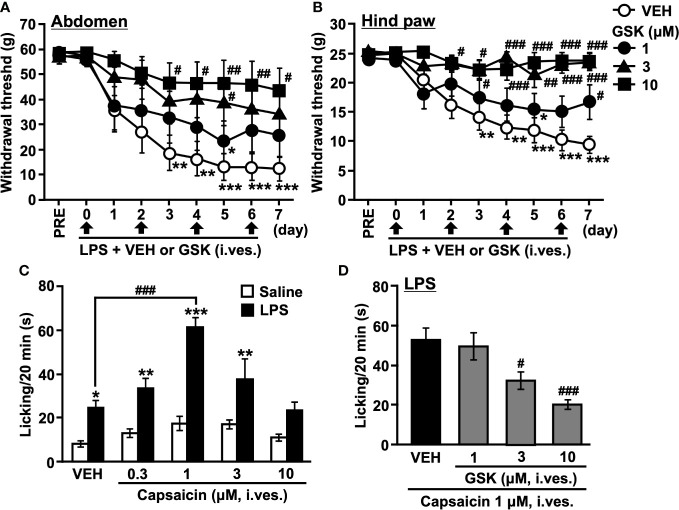
Repeated intravesical (i. ves.) instillation of GSK prevented the development of hypersensitivity to LPS-induced cystitis in rats. The von Frey filaments were applied to **(A)** the abdominal area close to the urinary bladder and **(B)** the plantar surface of the hind paw. The mechanical withdrawal threshold is presented over time. **(C)** Total time spent licking behavior evoked by (i) ves. instillation of capsaicin during the 20 min of the test on saline- and LPS-treated groups. **(D)** Total time spent licking behavior evoked by (i) ves. instillation of capsaicin during the 20 min of the test on LPS- (LPS + VEH) and GSK-treated groups (LPS + GSK). Data represent the mean ± SEM for eight rats. **(A, B)** *P < 0.05, **P < 0.01, ***P < 0.001 vs pre values. **(C, D)** *P < 0.05, **P < 0.01, ***P < 0.001 vs saline. ^#^ *P* < 0.05, ^##^ *P* < 0.01, ^###^ *P* < 0.001 vs vehicle (VEH).

In our previous study, repeated intravesical exposures to LPS caused chronic cystitis and induced persistent bladder hypersensitivity in rats. In vehicle-instilled rats, withdrawal threshold values in the abdomen and hind paw significantly decreased three days after the first LPS instillation and remained lower than the pre-value during the experiment (**Figures 2A, B**). The selective TRPV4 agonist GSK1016790A (GSK)-instilled rats showed significantly higher withdrawal threshold values in the abdomen and hind paw than the vehicle-instilled rats (**Figures 2A, B**).

Nociceptive behaviors, such as licking caused by capsaicin in the bladder, were counted to evaluate bladder nociception (**Figures 2C, D**). Compared to the saline-instilled group, the LPS-instilled group showed a significant increase in the duration of licking (**Figure 2C**). The licking behaviors were remarkable in the first 0-5 min and then gradually decreased. At lower doses (0.1-1 μM), intravesical capsaicin increased the licking time in a dose-dependent manner, and the most effective dose was 1 μM. In turn, higher doses of capsaicin (3-10 μM) decreased the licking time (**Figure 2C**). Based on these results, we selected 1 μM capsaicin and assessed the total licking time for 20 min in the following experiments. The significant increase in licking time that occurs with vehicle instillation may reflect pain during urine storage. Co-instillation of GSK (1-10 μM) with LPS reduced the increase in licking time induced by capsaicin in a dose-dependent manner compared to the LPS-instilled group (**Figure 2D**).

### TRPV4 inhibits the development of LPS-induced bladder overactivity

**Figure 3:**
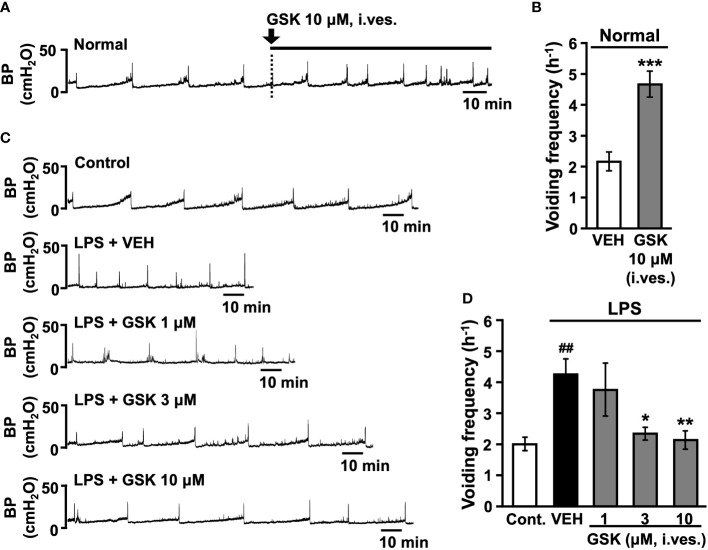
Effect of intravesical (i. ves.) instillation of GSK on normal or LPS-induced cystitis in conscious rats. **(A)** Representative continuous cystometrogram showing the effects of GSK bladder acute infusion in normal rats. **(B)** Changes in the voiding frequency of normal rats perfused with saline or GSK solution. **(C)** Representative continuous cystometrogram tracings from control, LPS- (LPS + VEH), and GSK-instillated group (LPS + GSK). **(D)** Changes in the voiding frequency in each group. Data represent the mean ± SEM from 5-8 rats. *P < 0.05, **P < 0.01, ***P < 0.001 vs vehicle (VEH). ^##^ *P* < 0.01 vs control. BP, bladder pressure.

Representative traces of cystometrograms obtained after acute intravesical infusion of GSK (10 μM) are shown in **Figure 3A**. Acute intravesical GSK (10 μM) *via* catheter infusion induced an increase in the voiding frequency compared to that before instillation (**Figures 3A, B**).

Representative traces of cystometrograms obtained after repeated intravesical instillation of saline (control) or LPS with vehicle or GSK (1-10 μM) are shown in **Figure 3C**. The vehicle-instilled group showed a significant increase in voiding frequency after LPS stimulation than the control group (**Figures 3C, D**). GSK-instilled (1-10 μM) group dose-dependently reduced the LPS-induced voiding frequency (**Figures 3C, D**). Intravesical instillations of GSK significantly reduced voiding frequency at doses of 3 and 10 μM compared with the vehicle group (**Figure 3D**).

### TRPV4 downregulates the LPS-induced production of inflammatory cytokines

**Figure 4:**
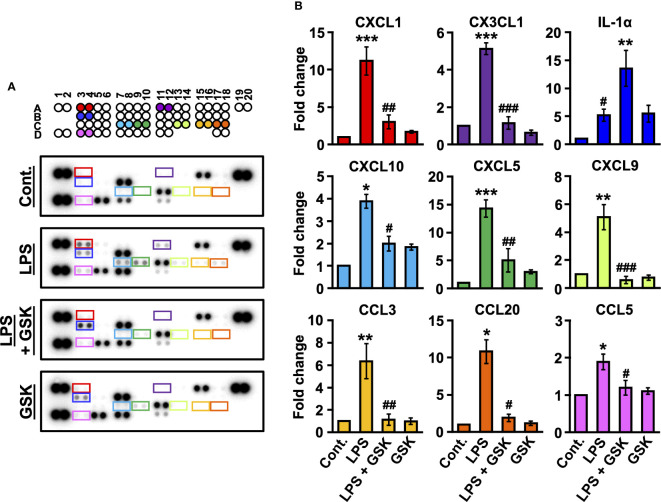
Changed secretion of LPS-induced inflammatory cytokines by GSK in rat bladder. **(A)** Raw images of the cytokine array for each group. **(B)** Fold change was calculated relative to the control. Data represent the mean ± SEM; *n* = 3 for each group. *P < 0.05, **P < 0.01, ***P < 0.001 vs control. ^#^ *P* < 0.05, ^##^ *P* < 0.01, ^###^ *P* < 0.001 vs LPS or LPS + GSK.

To determine whether the presence of GSK (10 μM) affected the production and secretion of cytokines, we analyzed the levels of inflammatory cytokines present in the rat bladder using a cytokine array. As shown in **Figure 4A**, after correcting for background intensity, nine cytokines/proteins were found to be significantly altered in the rat bladder after LPS treatment, including CXC chemokine ligands (CXCL1, CXCL5, CXCL9, and CXCL10), CC chemokine ligands (CCL3, CCL5, and CCL20), CX3C chemokine ligand 1 (CX3CL1), and interleukin-1α (IL-1α). Cytokine profiling data showed that a series of chemokines, including CXCL1, CXCL5, CXCL9, CXCL10, CCL3, CCL5, CCL20, and CX3CL1, were increased by LPS (**Figure 4B**). Compared to LPS alone, the addition of GSK (10 μM) significantly downregulated the production and secretion of these chemokines (**Figure 4B**). In contrast, the concurrent instillation of GSK (10 μM) significantly increased IL-1α levels compared to LPS alone (**Figure 4B**).

### TRPV4 changes macrophage polarization in the rat bladder

**Figure 5:**
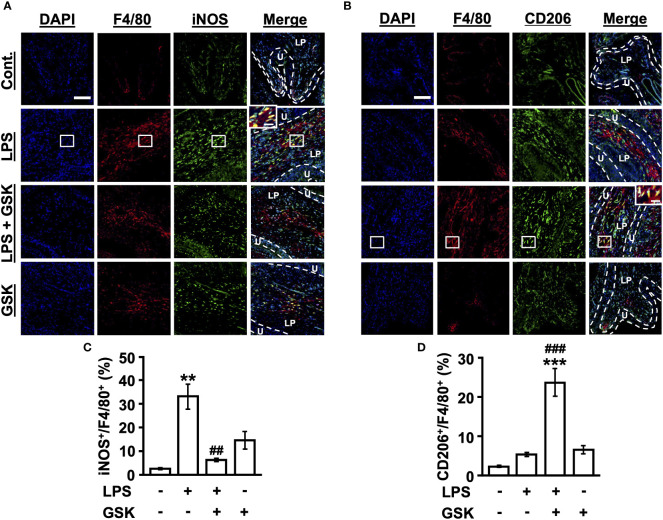
GSK regulates M1/M2 macrophage polarization in rat bladder. **(A)** Immunostaining of iNOS^+^F4/80^+^ macrophages in bladder tissues for each group. **(B)** Immunostaining of CD206^+^F4/80^+^ macrophages in bladder tissues for each group. Magnification 20× (object lens), scale bars, 100 μm. The boxed region from the merge is enlarged in the inset (scale bars, 20 μm). **(C)** Quantitative analysis of iNOS^+^ (M1 marker) cells as a percentage of total F4/80^+^ cells in rat bladder tissues. **(D)** Quantitative analysis of CD206^+^ (M2 marker) cells as a percentage of total F4/80^+^ cells in rat bladder tissues. Data represent the mean ± SEM; *n* = 4 for each group. **P < 0.01, ***P < 0.001 vs control. ^##^ *P* < 0.01, ^###^ *P* < 0.001 vs LPS. U, urothelium; LP, lamina propria.

First, we characterized M1 macrophages in the bladder tissue of rats *in vivo*. An increase in the number of iNOS^+^F4/80^+^ M1 macrophages below the epithelial layer was observed in LPS-treated rat bladders using immunofluorescence staining, which was suppressed by the activation of TRPV4 (**Figures 5A, C**). We next assessed whether GSK (10 μM) regulates M2 macrophage differentiation in response to LPS stimulation. We observed that the number of CD206^+^F4/80^+^ M2 macrophages increased in LPS + GSK-treated rat bladders (**Figures 5B, D**). GSK (10 μM) alone did not affect macrophage polarization (**Figures 5A–D**). Taken together, TRPV4 and LPS signals can inhibit M1 macrophage polarization and increase the induction of M2 polarization *in vivo*.

## Discussion

Our results show the potential benefit of the intravesical administration the selective TRPV4 agonist GSK for painful bladder hypersensitivity in a rat model of LPS-induced cystitis. Specifically, concurrent instillation of GSK into the rat bladder improved bladder tissue inflammation caused by LPS and suppressed bladder pain-related behaviors and voiding frequency. LPS stimulation induces higher levels of pro-inflammatory chemokines, whereas these chemokines are downregulated in the presence of GSK. The higher CD206^+^/F4/80^+^ ratios observed in bladder tissues activated in the presence of GSK suggest a change to an M2-type macrophage, whereas LPS induces an M1-type macrophage with a higher iNOS^+^/F4/80^+^ ratio. Taken together, these data suggest that interaction between the LPS signal and TRPV4 improves the internal environment of bladder tissue and induces downregulation of pro-inflammatory chemokines and macrophage phenotypic changes, leading to the resolution of inflammatory-associated painful bladder hypersensitivity.

In this study, we found increased inflammatory cell, including mast cells, infiltration into the submucosal and muscular layers in the bladder tissue of rats with LPS-induced IC/BPS, which is consistent with other studies in IC patients and various experimental models (27–30). Conversely, in LPS + GSK-treated rats, bladder inflammation was suppressed, and mast cell infiltration was reduced, suggesting that TRPV4 activity in LPS signaling suppresses inflammatory cell infiltration into the bladder. Furthermore, we found that concurrent instillation of GSK and LPS into the rat bladder prevented LPS-induced bladder pain and bladder hyperactivity. However, other studies have demonstrated opposite results, that is, improvement of bladder pain and overactivity in animal models of IC/BPS induced by LPS or CYP resulting from the administration of selective TRPV4 antagonists, which block the activation of this ion channel, or due to the deletion of the Trpv4 gene (20, 22, 31). TRPV4-KO mice show impaired development of bladder pain and overactivity after CYP, despite obvious signs of severe cystitis (20, 22). Our study showed that acute intravesical infusion of the selective TRPV4 agonist GSK caused an increase in bladder voiding frequency in normal rats. The activation of TRPV4 has been shown to induce ATP release, which triggers the afferent pathway *via* activation of the P2X3 receptor (16, 17, 22). Therefore, these data indicating that pharmacological inhibition or deletion of Trpv4 gene alleviates bladder pain and overactivity may be due to inhibition of this mechanism. In contrast, our data suggest that activation of TRPV4 may play a protective role in the inflammatory response of LPS by regulating the immune response. However, the bladder tissue from GSK alone-treated rats showed an inflammatory reaction with immune cells infiltration and an increased number of mast cells. In macrophages, TRPV4 has been demonstrated to exert both protective and detrimental effects to the host tissue, by facilitating bacterial clearance in infection while promoting parenchymal injury in sterile inflammation (32–36). Thus, TRPV4 activation may act pro-inflammatory in sterile inflammation or uninflamed bladder but may be protective against infectious inflammation. However, recent studies have shown that activation of protease-activated receptor (PAR)-2 by thrombin after LPS stimulation suppresses TRPV4 activity in macrophages and resolves lung injury (37). Similarly in LPS-induced acute lung injury model, TRPV4 also exacerbates the lung injury (38), and these LPS responses have been shown to be dependent on nuclear factor of activated T cells (NFAT) signaling *via* TRPV4-mediated Ca^2+^ influx (37, 38). Although the conflicting data on the role of TRPV4 in lung inflammation/injury, some consensus exists on the protective role of TRPV4 in macrophages (23, 25) and epithelial cells (21, 24). As a regulator of innate immunity and host defense, TRPV4 may sense mechanical changes in the extracellular environment during inflammation and regulate both pro- and anti-inflammatory effects.

In recently published data, urothelial cells of TRPV4-KO mice displayed LPS-induced enhancement of gene expression of pro-inflammatory cytokines *Il-6, Cxcl1, Cxcl2, and Tnf* (21). This is consistent with our data that TRPV4 activation suppressed LPS-stimulated inflammatory chemokines CXCL1, CXCL5, CXCL9, CXCL10, CCL3, CCL5, CCL20, and CX3CL1, suggesting that TRPV4 activation can have a negative regulatory effect on LPS-induced gene expression of some pro-inflammatory cytokines in the bladder. Cytokines and chemokines produced by bronchial epithelial cells can accumulate and activate various effector cells, including mast cells, neutrophils, monocytes, T cells, B cells, eosinophils, and dendritic cells in the airway (39). Furthermore, the co-culture of bronchial epithelial cells with mast cells elevates IL-6, CXCL1, and CXCL8 in bronchial epithelial cells (40). The etiology of IC/BPS involves an increase in bladder mast cells (41), which was also increased in LPS-induced IC/BPS model rats in this study. Mast cells produce a variety of cytokines and chemokines, including CXCR3 ligands CXCL9 and CXCL10, which attract neutrophils (42) and T cells (43) and partially contribute to bladder injury (44). Therefore, TRPV4 activity in LPS signaling may regulate the cytokines and chemokines produced by urothelial cells and downregulate the induction of further inflammatory mediators by suppressing mast cell activity. Previous studies have reported increased expression of several chemokines, such as CXCL1, CXCL9, CXCL10, and CCL5, in the bladder and urine of patients with IC/BPS with Hunner lesions (2, 45–47), suggesting that the anti-inflammatory effects of TRPV4 could be a potential therapeutic target for IC/BPS with Hunner lesions. However, the bladder tissue from GSK alone-treated rats showed an inflammatory reaction with immune cells infiltration and an increased number of mast cells, but no significant increase in chemokines. Since TRPV4 activation was reported to have no influence on the level of TLR4-dependent phosphorylation of NF-κB or its translocation to the nucleus, as observed with LPS stimulation (21), they might not have observed an increase in chemokines.

Alpizar et al., showed that LPS triggers an immediate response in intracellular Ca^2+^ in bladder and airway epithelial cells in a TLR4-independent manner, *via* the activation of TRPV4 (21, 24). In this regard, they concluded that LPS-induced activation of TRPV4 triggers signaling mechanisms that lead to immediate protective responses in epithelial cells and that this channel is a key player in innate defense responses. A protective role for TRPV4 has also been reported in immune cells, such as macrophages (23, 25) and microglia (48). Scheraga et al. demonstrated that macrophage TRPV4 is sensitized by extracellular matrix stiffness in the range of inflamed/fibrotic lungs and cooperates with LPS to mediate macrophage phenotypic changes (23, 25). This macrophage phenotypic change results in increased phagocytosis and secreted pro-resolution cytokines (decreased IL-1β, IL-6, CXCL1, and CXCL2, and increased IL-10). The current study also demonstrated that TRPV4 activation is important for the phenotypic change of M2 macrophages with upregulated CD206 in bladder tissues. LPS-stimulated rat bladders were found to have an increased expression of iNOS, an M1 macrophage-associated marker. Submucosal M1 macrophages are increased by LPS stimulation, resulting in higher levels of pro-inflammatory cytokines that define inflammatory T cell responses. Our results revealed that the T cell chemokines CCL3, CCL5, and CXCL10 were significantly abundant in the LPS-stimulated rat bladder. In addition, our results showed that concurrent treatment with GSK, a TRPV4 agonist, markedly reduced the expression of inflammatory cytokines and polarized macrophages toward the M2 phenotype. Although cytokine expression profiling in our study failed to detect some cytokines, M2 macrophages have high phagocytosis capacity and are associated with wound healing and resolution of inflammation by producing anti-inflammatory factors such as IL-10 and transforming growth factor-β (TGF-β) (49).

The mechanism of action of these anti-inflammatory/pro-resolution through TRPV4 in bladder tissues remains unclear. However, a recent study reported that TRPV4 modulates the LPS response in macrophages by switching from c-Jun N-terminal kinase (JNK) activation to p38 mitogen-activated protein kinase (MAPK) activation through dual-specificity phosphatase 1 (DUSP1) (25). The MAPK phosphatase DSUP1 has been identified as an important negative regulator of MAPK activity (50, 51), and DUSP1 induction response to LPS is reduced in the absence of TRPV4. This downregulation of phosphatase acts selectively to enhance JNK activation. This molecular switch, in the presence of TRPV4, mediates macrophage phagocytosis and downregulation of pro-inflammatory chemokine secretion in response to LPS. Future studies of this relationship should be conducted more directly using cultured urothelial cells and macrophages to confirm the molecular mechanisms of MAPK activation switching and macrophage M1/M2 polarization in the bladder.

Interestingly, our data indicated that the concurrent activation of TRPV4 markedly increased IL-1α more than LPS alone. Although IL-1α is known as a pro-inflammatory factor responsible for inducing inflammation (52, 53), it has been reported to work locally through another mechanism of neuroprotection and neurogenesis during post-stroke inflammation (54). Since IL-1α induces p38 MAPK phosphorylation/activation (55) and activated p38 MAPK strongly influences IL-1α expression (53), this may be associated with MAPK switching from JNK to p38. However, more studies are necessary to determine whether an increase in IL-1α leads to the resolution of inflammation, taking into account its negative effects.

## Conclusions

In summary, our study is the first to show that TRPV4 activation cooperates with LPS to induce reduced cellular immune responses, such as neutrophils and mast cells, and downregulation of inflammatory chemokines and phenotypic changes in macrophages, leading to the resolution of inflammation-related painful bladder hypersensitivity. Although pharmacological inhibition of TRPV4 has been proposed as a treatment for several experimental IC/BPS models, activation of TRPV4 function in response to infectious stimuli suggests that it may be a promising therapeutic approach not only for chronic inflammatory diseases, such as IC/BPS but also for various inflammatory diseases.

## Data availability statement

The original contributions presented in the study are included in the article/supplementary material. Further inquiries can be directed to the corresponding author.

## Ethics statement

The animal study was reviewed and approved by Committee of Animal Experiments, Tohoku Medical and Pharmaceutical University.

## Author contributions

MY conceived and designed the project, and wrote the manuscript. MY conducted and analyzed the von Frey experiments, histological stains and Immunostaining. MY and NT performed the capsaicin experiments, cystometry recordings and cytokine array. MY contributed to the data analyses. MY, CW, and HM contributed to the interpretation of data. All authors edited the manuscript. All authors contributed to the article and approved the submitted version.
